# Hemostatic Effect of Palliative Radiation Therapy in Preventing Blood Transfusions from Bleeding Occurring within Advanced Gastric Cancer

**DOI:** 10.1089/pmr.2021.0041

**Published:** 2021-12-22

**Authors:** Norio Mitsuhashi, Hajime Ikeda, Yoshitaka Nemoto, Mayumi Kuronuma, Masahiro Kamiga, Yoshinori Hiroshima

**Affiliations:** ^1^ Cancer Board Division, Radiation Therapy Center, Hitachinaka General Hospital, Hitachinaka, Ibaraki, Japan.; ^2^ Department of Palliative Care, amd Hitachinaka General Hospital, Hitachinaka, Ibaraki, Japan.; ^3^ Department of Gastroenterology, Hitachinaka General Hospital, Hitachinaka, Ibaraki, Japan.

**Keywords:** advanced gastric cancer, hemostatic effect, radiation therapy

## Abstract

**Purpose:**

To report the hemostatic effects of palliative radiation therapy (RT) for the prevention of blood transfusions (BT) in patients with advanced gastric cancer (AGC).

**Methods and Materials:**

Twenty-eight patients who received palliative three-dimensional conformal RT for hemostasis of gastric bleeding were retrospectively assessed in a study conducted in Japan. The median follow-up was 143.5 days. Changes in hemoglobin (Hb) levels were compared at the beginning of RT and four weeks later. Blood transfusion-free survival (BTFS) and overall survival (OS) were measured from the beginning of RT. Treatment toxicity was evaluated within 60 days of RT initiation.

**Results:**

No statistically significant decrease in Hb level was observed four weeks after RT. Twenty-eight patients did not receive BT within a month after RT, of whom three died within a month; 6/28 patients (21%) received BT at a median interval of 99.5 days following RT. The one-year BTFS and OS rates for all patients were 69% and 12%, respectively. The one-year BTFS was statistically significantly higher in 17 patients treated with a biologically effective dose (BED)_10_ of 39 Gy (30 Gy in 10 fractions) (78%) compared with six patients treated with a BED_10_ of 48 Gy (40 Gy in 20 fractions) (25%). Grade 1 and 2 nausea (*n* = 11) and a Grade 2 increase in alanine aminotransferase (*n* = 1) were observed. One patient died of Grade 5 hemorrhage.

**Conclusions:**

Palliative RT is an effective treatment to prevent BT for bleeding occurring within AGC. Specifically, a fractionation regimen of 30 Gy in 10 fractions (a BED_10_ of 39 Gy) has a more durable hemostatic effect and thus should be considered for better prognosis.

## Introduction

Gastric cancer was the third-most commonly occurring cancer in Japan (males, *n* = 72,617; females, *n* = 37,077) and the second leading cause of death for males and the third leading cause of death for females in 2019.^1^ The five-year survival rates for advanced gastric cancer (AGC) in 2019 were ∼50% for stage IIIA disease, 30% for stage IIIB disease, and 15% for stage IV disease.^1^ Local symptoms of AGC include pain, obstruction, and bleeding. The resulting anemia may cause fatigue and weakness. Repeated blood transfusions (BT) may be needed to maintain quality of life.

There are several treatment options for hemostasis, including surgical resection, endoscopic intervention, intravascular embolization, and radiation therapy (RT).^2,3^ RT is considered effective for achieving hemostasis following bleeding from AGC as it reduces the tumor burden while relieving symptoms.

Few studies have evaluated the hemostatic effects of palliative RT on bleeding from AGC.^4–12^ Kosugi et al. performed a national survey on the hemostatic effect of RT on bleeding from gastrointestinal and bladder cancer, reporting that hemostatic irradiation was rarely used and that optimal fractionation regimens remain unclear.^4^ This study aimed to report the role of RT in bleeding from AGC within our institutional experience.

## Materials and Methods

### Patients

We conducted a retrospective analysis of patients with AGC. Patients were recruited from our institutional cohort database (October 2015–June 2020) to report the hemostatic effects of palliative RT on gastric bleeding. Twenty-eight patients received palliative RT for hemostasis. Three patients with a previous partial gastrectomy for peptic ulcer disease or early gastric cancer were included in this study. All patients provided written informed consent for RT. As this study was a retrospective analysis of clinical outcomes, the institutional review board needed to be notified about the study, but approval for the study was not required.

This study enrolled 21 men and seven women (Table 1). The median age was 80 years (range 63–91); 43% of patients had poor performance status (3 or 4). The T stages were T1 (*n* = 1), T2 (*n* = 3), T3 (*n* = 8), and T4 (*n* = 16). A patient with T1 disease was classified as stage IV because para-aortic lymph node metastases (No. 16) were observed despite the early primary lesion; 15/28 patients had stage IV disease. Cancer originating from the gastric body was the most common cancer type (39%). All patients had been pathologically diagnosed with adenocarcinoma of the stomach. Eight had poorly differentiated adenocarcinoma.

**Table 1.** Demographic, Tumor, and Treatment Characteristics

Variable	No. of patients (GSC)	%
Gender		
Male	21 (3)	75
Female	7	25
ECOG PS		
1	10 (2)	36
2	6 (1)	21
3	9	32
4	3	11
T stage		
T1	1	4
T2	3	11
T3	8 (1)	28
T4	16 (2)	57
N stage		
N0	9 (2)	32
N1	5 (1)	18
N2	6	21
N3	8	29
Clinical stage		
II	8 (2)	29
III	5 (1)	18
IV	15	53
Pathological differentiation		
Moderate well differentiated adenocarcinoma	20 (3)	71
Poorly differentiated adenocarcinoma	8	29
Tumor location		
Cardia	4	14
Fundus	3 (1)	11
Body	11 (2)	39
Angular portion	4	14
Antrum	5	18
Pylorus	1	4
Blood transfusion		
Before RT	22 (1)	79
During RT^a^	10	36
After RT	8	29
None	5 (2)	18
Chemotherapy		
Before RT	15 (1)	54
During RT	3	11
After RT	4	14
None	12 (2)	43

^a^ Blood transfusion scheduled before the start of RT.

Clinical Stage, UICC TNM Classification of Malignant Tumor (8th Edition); ECOG-PS, Eastern Cooperative Oncology Group Performance Status; GSC, gastric stump cancer; RT, radiation therapy.

### Radiation therapy

Treatment planning and RT were performed after a four-hour fast and under shallow free-breathing conditions. Computed tomography (CT) for treatment planning was performed under three phases of respiration (normal breathing, hold shallow inspiration, hold shallow respiration) to determine the internal target volume (ITV). CT was performed on two consecutive days to determine the daily change in planning target volume (PTV).

Gross tumor volume (GTV) was determined using CT and endoscopic findings at the RT planning. In 23 patients, the partial stomach was determined as the GTV via enhanced CT images and/or metal markers placed on the cranial and caudal sites of the tumor (Table 2). Argon plasma coagulation and spraying of 10,000 U of thrombin were performed at clipping in four and three patients, respectively. The clinical target volume (CTV) encompassed the GTV at the time of the simulation. In the remainder of patients, for whom the extent of mass extension in the stomach could not be examined by enhanced CT or gastroscopy, the entire stomach was determined as the CTV. Regional lymph nodes were not included within CTV.

**Table 2.** Characteristics of Radiation Therapy

Variable	No. of patients (GSC)	%
CTV (stomach)		
Partial stomach	23 (3)	82
Entire stomach	5	18
Number of portals		
3	1	4
4	10	36
5	17 (3)	60
Total radiation dose		
20 Gy in 5 fractions	1	4
24 Gy in 12 fractions	1^a^	4
30 Gy in 10 fractions	17 (2)	60
34 Gy in 17 fractions	1^a^	4
36 Gy in 18 fractions	2^a^	7
40 Gy in 20 fractions	6 (1)	21
BED_10_		
39 Gy or less	19 (2)	68
>39 Gy	9 (1)	32

^a^ No. of patients who could not complete the scheduled RT.

(One of two patients who received 36 Gy in 18 fractions).

BED_10_, Biologically Effective Dose calculated by the Linear Quadratic Formula with an alpha/beta ratio of 10; CTV, clinical target volume.

**FIG. 1.**
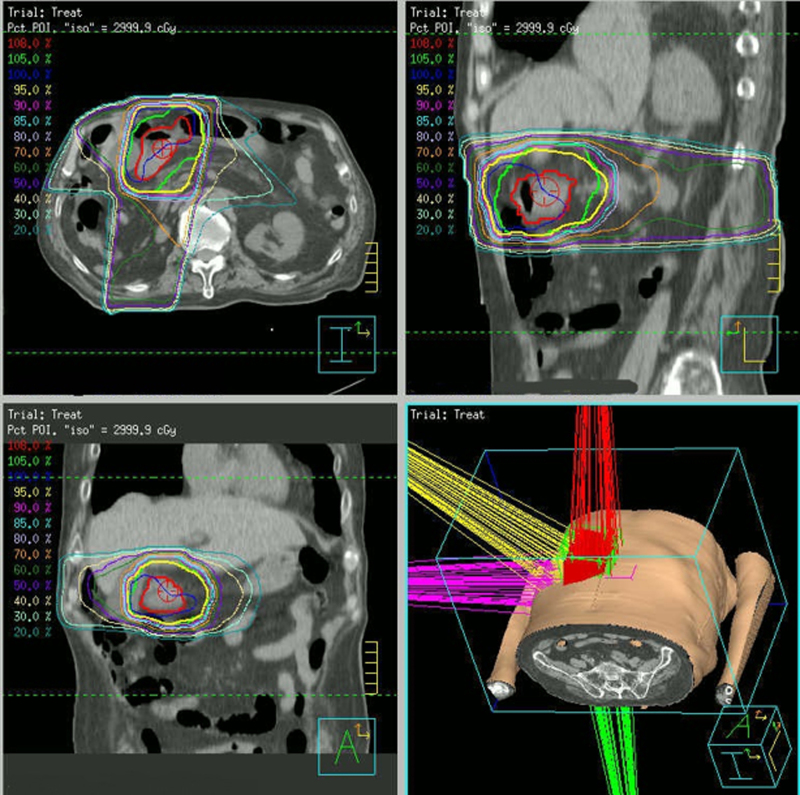
Representative 3DCRT planning with four static portals for gastric cancer originating from the gastric antrum. Case: Male (85 years.); Stage IIIA (T3N2M0). 3DCRT, three-dimensional conformal RT; RT, radiation therapy.

ITV was determined according to six respiratory phases within CT images over the course of two days. A PTV margin of 0.5–1.0 cm was used with three-dimensional conformal RT (3DCRT) for radiation delivery. Daily low-dose cone-beam CT was used for image guidance. All patients were treated with 3DCRT with six or 10 MV X-rays. Figure 1 shows a representative treatment plan.

The median total dose was 30 Gy (range: 20–40 Gy). A total dose of 30 Gy was delivered to 17/28 patients in 10 fractions. The biologically effective dose (BED), calculated using the linear quadratic formula, was used to compare the fractionation regimens.^13^ BED_10_ was calculated using an alpha/beta ratio of 10.

### Chemotherapy

Fifteen patients received chemotherapy (CH) before RT. The first-line CH regimens were S-1 (*n* = 6), S-1/cisplatin (*n* = 6), S-1/oxaliplatin (SOX) (*n* = 2), and S-1/cisplatin/trastuzumab (*n* = 1). Six patients received second-line CH comprising ramucirumab (RAM)/paclitaxel (PTX) (*n* = 3), RAM/nab-PTX (*n* = 2), and camptothecin-11 (CPT-11) (*n* = 1). Nivolumab was administered to two patients as third-line CH. Three patients received concurrent chemoradiotherapy (CRT) (S-1, *n* = 2 and CPT-11, *n* = 1). CH was administered after RT in four patients (S-1, *n* = 2; G-SOX/trastuzumab, *n* = 1; trifluridine and tipiracil hydrochloride, *n* = 1). Twelve patients did not receive CH during the clinical course.

### BT and hemoglobin levels

BT was performed in 22 patients before RT; 18/28 patients did not receive BT during the RT period. The remaining 10 patients received BT (which had been scheduled before the start of RT) later, because RT was performed as soon as possible to achieve hemostasis.

Hemoglobin (Hb) levels were measured two, three, four, and six weeks after RT (*n* = 5, *n* = 1, *n* = 21, and *n* = 12, respectively). Hb levels could not be measured in one patient who was transferred to hospice immediately after RT. Hb levels were measured the day after BT in 10 patients who received BT during RT. Ten patients had Hb measurements conducted at both four and six weeks after RT.

### Evaluation and statistical analyses

The follow-up period was calculated from the start of RT, and a prognosis survey was conducted in March 2021. No patient was lost to follow-up. The median follow-up period was 143.5 days (range 18–663 days). Seven patients were alive at the end of follow-up.

Changes in Hb levels were compared at the start of RT and four weeks after the end of RT for patients who did not receive BT during RT. In patients who received BT during RT, changes in Hb levels were compared at the end of BT and four weeks after RT. In patients who had Hb measurements conducted at both four and six weeks after RT, changes in Hb levels were also compared at four and six weeks after RT. Statistical differences were compared using paired *t* tests. RT was defined as effective if the patients did not receive BT for more than one month after RT.

Blood transfusion-free survival (BTFS) and overall survival (OS) were measured from the start date of RT and assessed using the Kaplan–Meier method. Scheduled BT during RT was not considered in the calculation of BTFS time among 10 patients.

BTFS and OS were calculated by clinical stage, T stage, CH, and BED_10_. The log-rank test was used for statistical comparisons. Patients without BT were censored at the time of death.

*p*-Values <0.05 were considered statistically significant.

Treatment toxicities were evaluated within 60 days of RT initiation according to the CTCAE-v5.

## Results

### Completion of RT

Twenty-five out of 28 patients completed the scheduled RT. Two patients discontinued RT at 60% and 85% of the planned total dose, respectively, due to a worsening general condition. One patient was transferred to hospice at 90% of the planned total dose.

### Changes in Hb levels

**FIG. 2.**
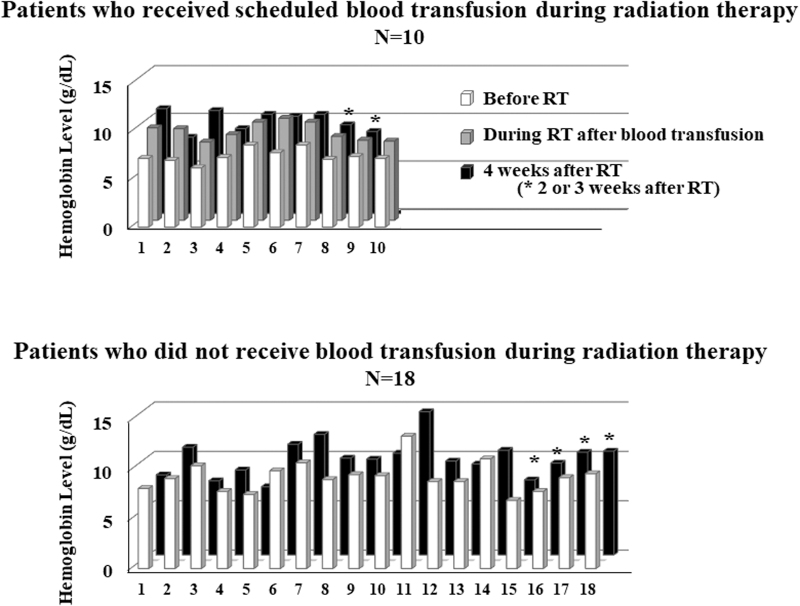
Changes in hemoglobin levels for each patient due to RT. Upper figure: Patients who received scheduled blood transfusion during RT. Lower figure: Patients who did not receive blood transfusions during RT.

**FIG. 3.**
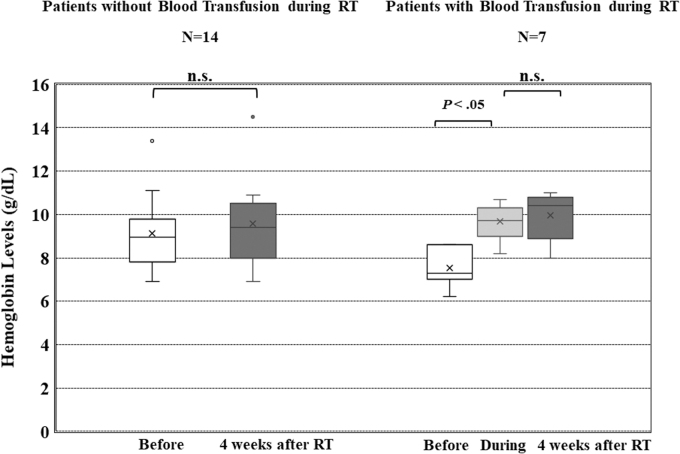
Box and whisker plots of the distribution of Hb levels among patients receiving RT. Left figure: Patients who did not receive blood transfusions during RT and who had their Hb levels measured four weeks after RT (before RT vs. after RT; *p* > 0.05). Right figure: Patients who received scheduled blood transfusions during RT and had their Hb levels measured four weeks after RT (before RT vs. during RT; *p* < 0.05, during RT vs. after RT; *p* > 0.05). Hb, hemoglobin.

Figure 2 shows changes in Hb levels due to RT for 10 patients who received scheduled BT during RT and for 18 patients who did not receive BT during RT. The mean Hb ± standard deviation (SD) was 9.5 ± 1.5 g/dL before RT and 9.9 ± 2.0 g/dL four weeks after RT for 14 patients who did not receive scheduled BT during RT (Fig. 3). This difference was not statistically significant. The mean Hb ± SD was 7.5 ± 0.9 g/dL before RT, 9.7 ± 0.9 g/dL after BT, and 10.0 ± 1.1 g/dL four weeks after RT for seven patients who received BT during RT. No statistically significant changes in Hb levels were observed between the day after BT and four weeks after RT. The mean Hb ± SD at four and six weeks after RT in 10 patients were 9.9 ± 1.4 g/dL and 10.0 ± 0.9 g/dL, respectively (*p* > 0.05).

### BT after RT

No patients received BT within a month after RT; three patients died during this interval. Six patients received BT with a median interval of 99.5 days from RT to BT (range 52–140 days). Thus, RT was found to be effective. The median follow-up for patients who received BT after RT was 127.5 days (range 91–266 days), while that of patients who did not receive BT after RT was 157.5 days (range 19–663 days). Four of 16 patients (25%) with T4 tumors received BT after RT, as did 2/12 patients (17%) with T1–3 tumors; 2/6 patients (33%) who received BT after RT were treated with concurrent and/or adjuvant CH, as were 2/22 patients (9%) who did not receive BT after RT.

No patients had hematemesis after RT during follow-up. It was not possible to assess the number of patients who developed melena after RT because most patients were administered iron supplementation.

### Survival

The one-year BTFS and OS rates in all patients were 69.4% and 12.1%, respectively.

**FIG. 4.**
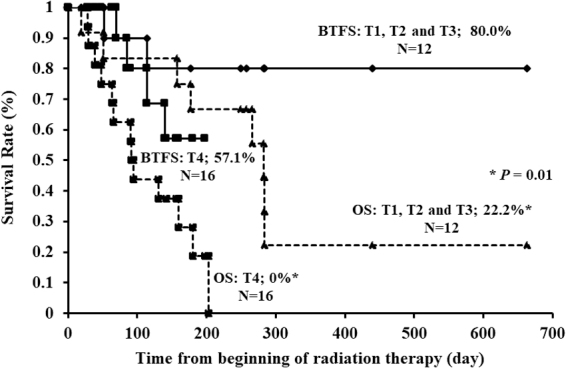
Kaplan–Meier curves of BTFS and OS after starting RT, stratified by T stage. BTFS: T1, T2, and T3 versus T4 (80.0% vs. 57.1%; *p* = 0.36). OS: T1, T2, and T3 versus T4 (22.2% vs. 0%; *p* = 0.01). BTFS, blood transfusion-free survival; OS, overall survival.

Figure 4 shows the BTFS and OS curves of all patients by T stage. There was no statistically significant difference in BTFS rate between patients with T1–3 tumors and those with T4 tumors (*p* = 0.36). BTFS rate was not statistically significantly different between patients with stage II/III disease and those with stage IV disease (*p* = 0.86).

**FIG. 5.**
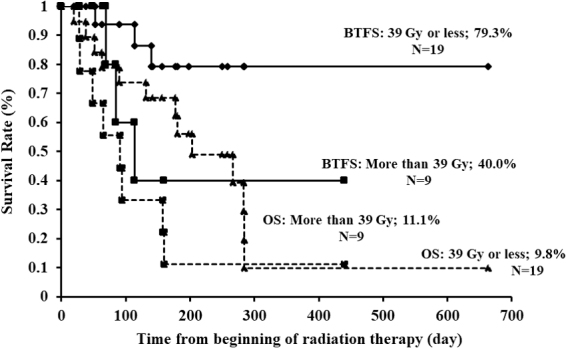
Kaplan–Meier curves of BTFS and OS after starting RT (BED_10_ ≤39 Gy or >39 Gy). BTFS: ≤39 Gy vs. >39 Gy (79.3% vs. 40.0%; *p* = 0.09). OS: ≤39 Gy vs. >39 Gy (9.8% vs. 11.1%; *p* = 0.09). BED, biologically effective dose.

**FIG. 6.**
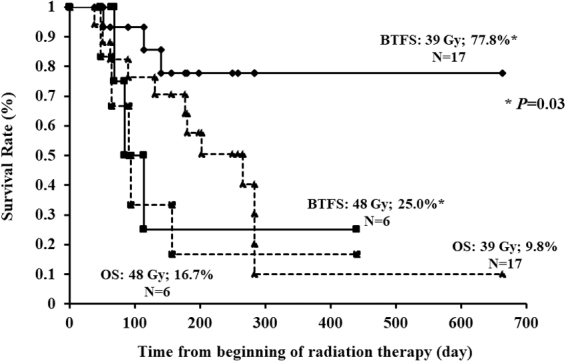
Kaplan–Meier curves of BTFS and OS after starting RT (BED_10_ = 39 Gy [30 Gy in 10 fractions] and 48 Gy [40 Gy in 20 fractions]). BTFS: 39 Gy versus 48 Gy (77.8% vs. 25.0%; *p* = 0.03). OS: 39 Gy versus 48 Gy (9.8% vs. 16.7%; *p* = 0.32).

Figure 5 shows the BTFS and OS curves of patients who received RT, stratified according to BED_10_ values ≤39 Gy or >39 Gy. There was no statistically significant difference in the one-year BTFS rates (*p* = 0.09). BTFS rates at one year were 77.8% and 25.0%, respectively, in patients treated with a BED_10_ of 39 Gy (30 Gy in 10 fractions) or with a BED_10_ of 48 Gy (40 Gy in 20 fractions) (*p* = 0.03) (Fig. 6).

**FIG. 7.**
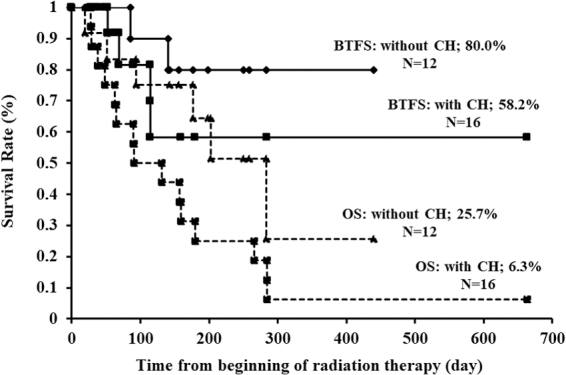
Kaplan–Meier curves of BTFS and OS after starting RT, stratified by CH. BTFS: RT alone versus CH + RT (80.0% vs. 58.2%; *p* = 0.28). OS: RT alone versus CH + RT (25.8% vs. 6.3%; *p* = 0.11). CH, chemotherapy.

The one-year BTFS rate in patients who did not receive CH was not statistically significantly different from that of patients who received CH (*p* = 0.28) (Fig. 7).

### Toxicity

Grade 1 and 2 nausea was observed in nine and two patients, respectively, including one patient who received concurrent CH. A transient Grade 2 increase in alanine aminotransferase two months after RT was observed in one patient who did not receive CH. One patient with a T3 tumor detected in the angular portion of the stomach did not receive CH and died from a massive Grade 5 gastric hemorrhage.

## Discussion

### Evaluation of RT

Many reports have established the criteria for the hemostatic effect of RT when BT is not required for more than four weeks.^5–9,14^ In most of the previous studies, the hemostatic effect of RT ranged from 50% to 80%, with a median duration of response in the range of 1.5–11.4 months.^5–9,11,14^ Tey et al. reported an overall pooled response rate for bleeding of 74% in their review article.^12^ In our case series, we observed that the hemostatic effect of RT lasted for more than one month in the 25 patients who survived; three patients died within a month (without BT). Thus, hemostatic events were observed in all survivors more than one month after RT. The median duration of the hemostatic effect was 3.8 months.

The six-month BTFS rate in patients with T4 tumors was inferior to that in patients with T1-T3 tumors; 4/6 patients who received BT after RT had T4 tumors. This difference was not statistically significant, but a previous study reported that T4 tumors had a trend toward inferior local control against bleeding.^5^

Asakura et al. reported that patients treated with concurrent CRT had a statistically significantly lower rebleed rate than those treated with RT alone, although Grade 3 or higher adverse effects, which were not observed among the patients treated with RT alone, were observed in those treated with CRT.^9^ No statistically significant difference in the one-year BTFS rate was observed between patients who did or did not receive CH.

### Tumor bed effect

RT has the added advantage of shrinking the tumor burden, in contrast to other palliative options for bleeding from AGC. Haveman et al. reported a considerable recovery of the tumor bed effect (TBE) after a radiation dose of 20 Gy at 180 days after irradiation.^15^ Milas et al. reported that TGF was induced by doses and fractionation schedules of RT commonly used in the clinic.^16,17^ TBE was induced by the doses and fractionation schedules of RT used in our patients to suppress the tumor regrowth accompanied with inhibition of the growth of new blood vessels for at least three to six months following RT.^18^ Hence, the hemostatic effect may have been sustained in later periods due to the shrinkage of tumor burden.

### Biologically effective dose

Previous studies have reported no clear correlations between BED_10_ and the hemostatic effect of RT within a wide variety of RT doses and fractionation regimens. Studies have reported higher hemostatic effects with a BED_10_ ≥50 Gy^8^ or ≥41 Gy or more,^5^ as well as general linear relationships between BED_10_ and hemostatic effects.^6^ Recent studies have demonstrated that short course RT with less than BED_10_ of 39 Gy (BED_10_ = 28 Gy; 20 Gy in 5 fractions, BED_10_ = 14.4 Gy; a single 8 Gy fraction), which is a more suitable option for patients with limited life expectancy, has a similar hemostatic effect as longer course RT with more than BED_10_ of 39 Gy, which has a risk of increased interruptions of RT and/or prolongation of hospital stay.^14,19,20^

In our study, the one-year BTFS of patients treated with a BED_10_ of 39 Gy (30 Gy in 10 fractions) was statistically significantly improved compared with that of patients treated with a BED_10_ of 48 Gy (40 Gy in 20 fractions). This study is retrospective and selection bias in patients cannot be ruled out. However, it can be considered that the hemostatic effect of 30 Gy in 10 fractions was not inferior to that of 40 Gy in 20 fractions. Considering the poor prognosis of patients with AGC, a fractionation regimen of 30 Gy in 10 fractions over two weeks should be selected in preference to that of 40 Gy in 20 fractions over the course of four weeks.

BED_10_ does not consider the overall treatment time. Even if 30 Gy was delivered in 10 fractions within two weeks or if 30 Gy was delivered in 10 fractions within four weeks, the BED_10_ was calculated as the same value (39 Gy). The biological effects of both irradiation schedules were the same; thus, the overall treatment time could be reduced, and the biological effect could be increased. If there is a waste dose in which the tumor cells are not damaged up to a certain dose because of the recovery potential for irradiation damage, a larger fraction dose will increase the radiation damage.

A larger fraction dose was more effective for adenocarcinoma as RT is considered less effective for adenocarcinoma than for squamous cell carcinoma, irrespective of the organ of origin.^12,19,21,22^ Hashimoto et al. recommended a fraction dose of 2.5 Gy (40 Gy in 16 fractions); a fraction dose of 3 Gy was more effective in our study. A larger fraction dose has been found to be more effective for adenocarcinoma of the stomach.^8^ RT with 30 Gy in 10 fractions (BED_10_ = 39 Gy) is considered an adequate treatment for bleeding from AGC, especially in patients with a poor prognosis.^9^

Although the role of dose escalation for improved tumor control remains to be established, the homeostatic effect should be evaluated within prospective studies of RT with hypofractionation, which has a shorter overall treatment time.^10,14,23^

### Toxicity

Grade 1 and 2 toxicities are very common and have been reported in up to 100% of patients, especially those with Grade 1–2 fatigue. Many previous studies only reported on toxicities of Grade 3 and above due to the self-limiting nature of Grade 1–2 toxicities. Asakura et al.^9^ reported that most patients develop minimal toxicity. Among patients receiving concurrent CRT, one patient developed Grade 3 nonhematological toxicity and five patients had Grade 3–4 hematological toxicity; no patients treated with RT alone developed Grade 3 or higher adverse events.^9^

In our study, it was not easy to assess the underlying mechanisms for gastrointestinal Grade 1 or 2 toxicities (e.g., BT, analgesia, CH, tumor progression). Grade 1–2 toxicities were classified as RT-induced adverse events in the current study. The frequency of adverse events in patients treated with CRT was higher in all reports compared with those treated with RT alone.

Toxicities were acceptable in patients treated with RT alone. Grade 3–4 acute toxicities were observed in up to 15% of patients treated with RT alone and in up to 25% of patients treated with CRT.

### Strengths and limitations

It was very difficult to create an objective parameter to evaluate the hemostatic effects of palliative RT for bleeding from AGC. Theoretically, the hemostatic effects of RT could be confirmed by direct observation of the bleeding lesion through an endoscopic approach in combination with measurement of Hb levels; however, it is practically difficult to repeatedly perform endoscopy on patients in poor general condition. There are no accurate measurable parameters to evaluate the effectiveness of RT for gastric cancer bleeding that can replace direct observation using an endoscope. It is difficult to determine appropriate objective parameters to assess the hemostatic efficacy of palliative RT for bleeding from AGC due to the following factors.

(1) Baseline Hb level differs by sex, age, comorbidity, and CH; thus, it is difficult to set a transient Hb as a standard evaluation tool for determining treatment outcomes. (2) The Hb level at the start of RT depends on whether BT was administered. (3) Hb levels requiring BT differ depending on the general condition of the patient and on whether CH was administered. (4) Many different doses and fractionation schedules for RT were used. (5) Different regimens of CH, analgesics, and steroids, which affect health outcomes and adverse events, are combined with RT. (6) There is currently no established patient-reported outcome that can provide valuable evidence to inform shared decision making for palliative interventions.^24^ (7) Researcher bias, leading to overestimation of therapeutic effects and underestimation of adverse effects, may affect the evaluation of results in retrospective studies.

## Conclusion

Twenty-eight patients with AGC received palliative RT with a median BED_10_ of 39 Gy for hemostasis of gastric bleeding. No patients received BT within one month after RT, and a statistically significant decrease in Hb was not observed one month after RT.

We found that RT with 30 Gy in 10 fractions (BED_10_ = 39 Gy) effectively treated bleeding from AGC, especially in patients with poor prognosis; acute adverse effects above Grade 3, with the exception of one case of Grade 5 gastric hemorrhage, were not observed. Therefore, we conclude that palliative RT is an effective and safe treatment for preventing repeated BTs due to bleeding from AGC.

## Data Sharing

The data used for this study, although not available in a public repository, will be made available to other researchers upon reasonable request.

## Abbreviations Used

3DCRT: three-dimensional conformal RT
AGC: advanced gastric cancer
BED: biologically effective dose
BT: blood transfusions
BTFS: blood transfusion-free survival
CH: chemotherapy
CPT-11: camptothecin-11
CRT: chemoradiotherapy
CT: computed tomography
CTV: clinical target volume
ECOG PS: Eastern Cooperative Oncology Group Performance Status
GSC: gastric stump cancer
GTV: gross tumor volume
Hb: hemoglobin
ITV: internal target volume
OS: overall survival
PTV: planning target volume
PTX: paclitaxel
RAM: ramucirumab
RT: radiation therapy
SD: standard deviation
SOX: oxaliplatin
TBE: tumor bed effect
